# Prestroke physical activity could influence acute stroke severity (part of PAPSIGOT)

**DOI:** 10.1212/WNL.0000000000006354

**Published:** 2018-10-16

**Authors:** Malin Reinholdsson, Annie Palstam, Katharina S. Sunnerhagen

**Affiliations:** From the Department of Clinical Neuroscience (M.R., A.P., K.S.S.), Institute of Neuroscience and Physiology, Sahlgrenska Academy at University of Gothenburg; Department of Occupational Therapy and Physiotherapy (M.R.), Sahlgrenska University Hospital, Gothenburg, Sweden.

## Abstract

**Objective:**

To investigate the influence of prestroke physical activity (PA) on acute stroke severity.

**Methods:**

Data from patients with first stroke were retrieved from registries with a cross-sectional design. The variables were PA, age, sex, smoking, diabetes, hypertension and statin treatment, stroke severity, myocardial infarction, new stroke during hospital stay, and duration of inpatient care at stroke unit. PA was assessed with Saltin-Grimby's 4-level Physical Activity Level Scale, and stroke severity was assessed with the National Institutes of Health Stroke Scale (NIHSS). Logistic regression was used to predict stroke severity, and negative binomial regression was used to compare the level of PA and stroke severity.

**Results:**

The study included 925 patients with a mean age of 73.1 years, and 45.2% were women. Patients who reported light or moderate PA levels were more likely to present a mild stroke (NIHSS score 0 to 5) compared with physically inactive patients in a model that also included younger age as a predictor (odds ratio = 2.02 for PA and odds ratio = 0.97 for age). The explanatory value was limited at 6.8%. Prestroke PA was associated with less severe stroke, and both light PA such as walking at least 4 h/wk and moderate PA 2–3 h/wk appear to be beneficial. Physical inactivity was associated with increased stroke severity.

**Conclusions:**

This study suggests that PA and younger age could result in a less severe stroke. Both light PA such as walking at least 4 h/wk and moderate PA 2–3 h/wk appear to be beneficial.

Stroke is the second leading cause of disability-adjusted life-years lost worldwide after ischemic heart disease.^1^ Ten modifiable risk factors are said to be associated with 90% of the causes of all stroke: history of hypertension, physical inactivity, high apolipoprotein, unhealthy diet, higher waist-to-hip ratio, psychosocial stress, current smoking, cardiac causes, alcohol consumption, and diabetes mellitus.^2^ Physical inactivity causes morbidity, mortality, and major economic burden worldwide. However, although physical activity (PA) is a well-known, self-monitored, cost-efficient method to increase health in individuals of all ages, physical inactivity is a global pandemic.^3^ Previous research in animals has shown that PA before stroke has a neuroprotective effect leading to less severe stroke with fewer neurologic deficits^4,5^ and better recovery.^5^ Human studies have, however, been less consistent. Some studies have shown that PA before stroke is associated with milder neurologic symptoms after stroke,^6–10^ whereas another showed no effect.^11^

The objective was to investigate the influence of prestroke PA, age, sex, smoking, diabetes, and protective treatments such as statin and hypertension treatments on acute stroke severity. We hypothesized that prestroke PA would positively influence acute stroke severity.

## Methods

The data used in this study were based on the Physical Activity Pre-Stroke In GOThenburg population, with data from the Swedish Stroke Register (Riksstroke)^12,13^ and the local stroke register, Väststroke. The personal identification numbers used in Sweden allow for linkage between registries, and anonymous data were received (linkage was performed by the Register service at the National Board of Health and Welfare). The reason for combining 2 registries was that one register could not deliver all variables. The study was a cross-sectional, nonexperimental study in which data on PA were collected retrospectively.

### Population

We extracted clinical data from 2,233 patients (1,158 men and 1,075 women) with stroke who were admitted to Sahlgrenska University Hospital from November 1, 2014, to April 30, 2016. The inclusion criteria were first-time stroke, receipt of care at stroke units at Sahlgrenska University Hospital in Gothenburg, and assessment with measures of stroke severity and PA. The exclusion criteria were former stroke or incomplete data.

### Descriptive variables

The *ICD-10* was used to identify patients with diagnoses of either intracerebral hemorrhage or ischemic stroke. Information about the number of days of inpatient care at the stroke unit and occurrence of new stroke or myocardial infarction during inpatient stay were retrieved from the registries.

### Dependent variable

Stroke severity at hospital admittance was assessed with the National Institutes of Health Stroke Scale (NIHSS; 0–42), for which a higher score indicates greater stroke severity.^14^ The cutoff scores were mild stroke (0–5), moderate stroke (6–14), severe stroke (15–24), and very severe stroke (≥25).^15^ The NIHSS was dichotomized into mild stroke (NIHSS score 0–5) and moderate to severe stroke (NIHSS score 6–42) in the regression analyses.

### Selection of independent variables

The independent variables selected were PA, age, sex, smoking, diabetes, and protective treatments such as statin or hypertension treatments. Prestroke PA was assessed with the Saltin-Grimby Physical Activity Level Scale (SGPALS).^16,17^ The SGPALS is a self-reported 4-level scale with the following levels: (1) physically inactive, (2) some PA for at least 4 h/wk (light PA), (3) regular PA and training for at least 2–3 h/wk (moderate PA), and (4) regular hard physical training for competition sports several times per week (vigorous PA).^16,17^ Patients were asked about their PA at their first encounter with a physical therapist at the stroke unit with the following question: “How much did you move or exercise during leisure time before stroke? Try to estimate an average.” The physical therapist asked the patient to be precise about the previous activity level with the help of follow-up questions about intensity, duration, and type of activity, and relatives were asked to confirm the PA level whenever needed. The SGPALS was dichotomized into physically inactive patients (level 1) and light, moderate, and vigorous physically active patients (level 2–4) in the regression analyses. The SGPALS was divided into 3 groups (levels 3 and 4 were merged) to discover differences between different levels of PA in negative binomial regression analyses.^17^

### Data analyses

IBM SPSS Statistics 24 was used for statistical analyses. Descriptive data are presented as mean with SD for continuous variables or number (n) and percentage (%) for categorical variables. The significance level was adjusted to *p* < 0.002 according to the Bonferroni correction. Differences between patients included in the study and patients with missing assessments (NIHSS and SGPALS) were analyzed with independent samples *t* tests for age and χ^2^ tests for sex. Nonparametric statistics were used because of the non-normal distribution of the data.

Negative binomial regression analyses with log link were performed to explore relationships between the levels of prestroke PA (SGPALS) and stroke severity (NIHSS). The NIHSS was defined as the dependent variable (continuous scale), and the SGPALS score divided into 3 levels were defined as factors.

Logistic regression was used to assess the effect of a set of predictors (PA, age, sex, smoking, diabetes, and protective treatments such as statin or hypertension treatments) on the dependent variable, defined as stroke severity (NIHSS). The following steps for the multiple regression model building were followed.^18,19^

1. Cross-tabulation was performed between each independent variable and the dependent variable to detect small or redundant groups, followed by dichotomization when needed.
2. Logistic regression (univariate analyses) was performed separately between each independent variable and the dependent variable.
3. Logistic regression (multivariate analysis) was performed backward with the remaining independent variables with *p* < 0.25. The least significant variables were removed one at a time until a model with significant independent variables was found.
4. The predictor model with the fewest independent variables was chosen, and Nagelkerke's^20^ determination coefficient, R^2^, was registered.

The goodness of explanatory variables was calculated with the Hosmer and Lemeshow test, and the area under the curve in a receiver operating characteristic curve was computed. A 2-way analysis of variance (ANOVA) was conducted to explore the possible interaction between independent variables in the final model on stroke severity (NIHSS).

### Standard protocol approvals, registrations, and patient consents

The Swedish Data Protection Authority allows handling of data generated within the framework of quality registers as an exception from the general rule of patient consent in view of public interest because it provides improvement of the quality of care and treatment. Furthermore, according to the Personal Data Act (Swedish law No. SFS 1998:204), no informed consent is needed to collect data from medical charts for clinical purposes and quality control. The study was approved by the Regional Ethical Board of Gothenburg on May 4, 2016 (registration number: 346-16).

### Data availability

Data from registries are subject to the Personal Data Act (Swedish law No. SFS 1998:204). Data may be available to researchers upon request, after review of secrecy (contact the author ks.sunnerhagen@neuro.gu.se). According to the Swedish regulation (epn.se/en/start/regulations/), the permission to use data can only be according to application and approval from the ethical board.

## Results

Of the 2,233 patients admitted with stroke from November 1, 2014, to April 30, 2016, 1,308 patients were excluded (figure).

**Figure:**
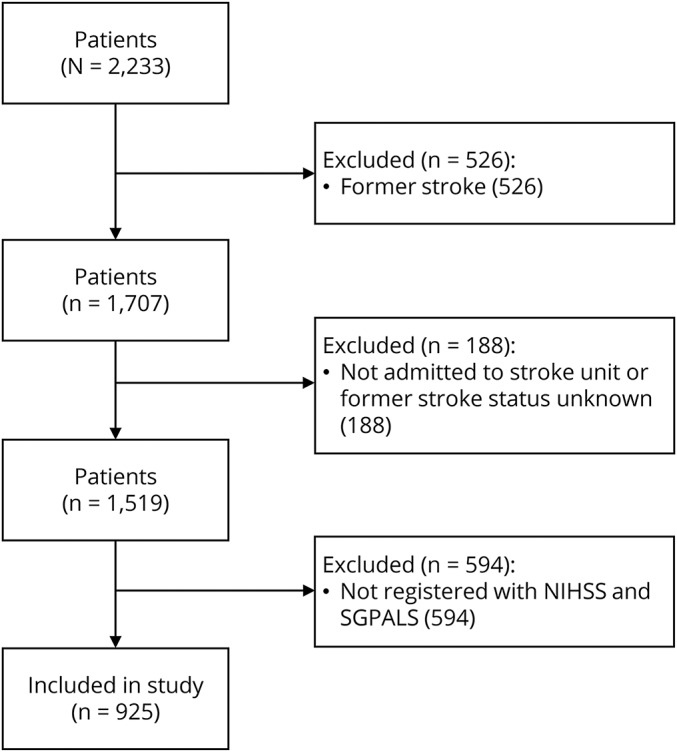
Flowchart of participants to describe the included study population NIHSS = National Institutes of Health Stroke Scale. SGPALS = Saltin-Grimbys Physical Activity Level Scale.

The final sample included 925 patients with stroke, with a mean age of 73 years (range 20–104) and 45.2% women. Ischemic stroke was the most common cause of stroke (93.8%), and 5.8% had cerebral hemorrhage. A majority of the patients were assessed as having a mild stroke (79.8%) and 34.2% had 0 points on the NIHSS after admission to hospital. The cutoff scores for the NIHSS were mild stroke (0–5), moderate stroke (6–14), severe stroke (15–24), and very severe stroke (≥25).^15^ Half of the population (52.0% of patients) reported that they were physically inactive before their stroke (table 1).

**Table 1:**
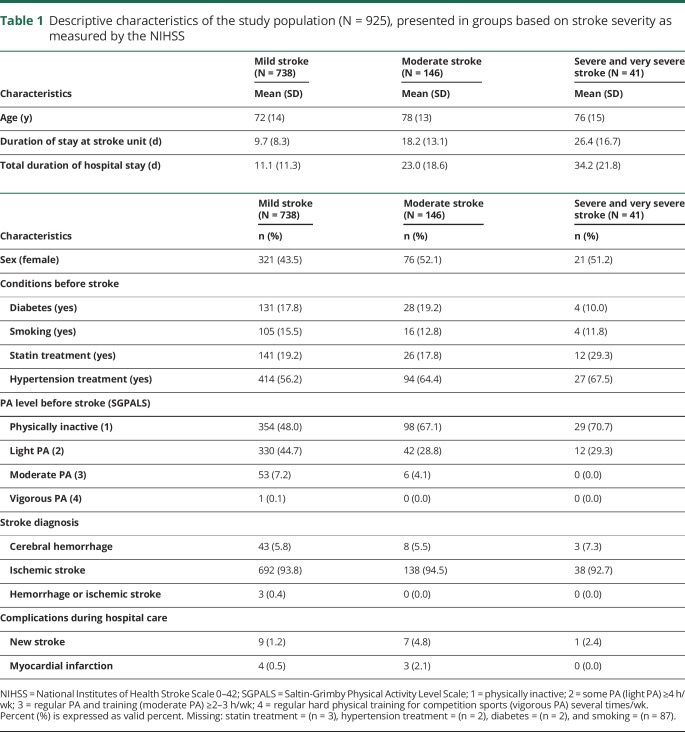
Descriptive characteristics of the study population (N = 925), presented in groups based on stroke severity as measured by the NIHSS

Excluded patients with missing NIHSS and SGPALS assessments were compared with patients included in the study. The excluded patients were older with a mean age of 76 years (*p* = 0.001) and contained more women (53.5%; *p* = 0.001) than the included group.

The physically inactive group had more severe stroke compared with both the light PA group (level 2; *p* < 0.001) and the moderate PA group (level 3–4; *p* < 0.001) when analyzed with negative binomial regression. There was no significant difference between the light PA group (level 2) and the moderate PA group (levels 3 and 4) on stroke severity as measured with the NIHSS (*p* = 0.140) (table 2).

**Table 2:**
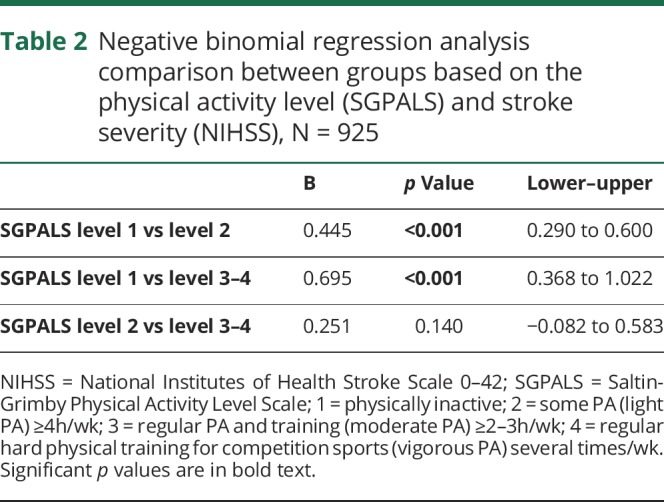
Negative binomial regression analysis comparison between groups based on the physical activity level (SGPALS) and stroke severity (NIHSS), N = 925

After analysis with univariate logistic regression, the variables PA, age, sex, and hypertension treatment (*p* < 0.25) were included in a multivariate analysis. In the multivariate analysis, the variables sex and hypertension were excluded. In the final model, PA and age remained. Patients who had been physically active before their stroke and were younger in age were more likely to have a mild stroke (odds ratio = 2.02 for PA and odds ratio = 0.97 for age). The model predicted 6.8% of stroke severity (table 3). There was no interaction effect between age and prestroke PA (*p* = 0.166) in a 2-way ANOVA analysis. Patients were divided into 4 groups according to their age (18–45 years, 46–65 years, 66–80 years, and 81–104 years), and prestroke PA was dichotomized.

**Table 3:**
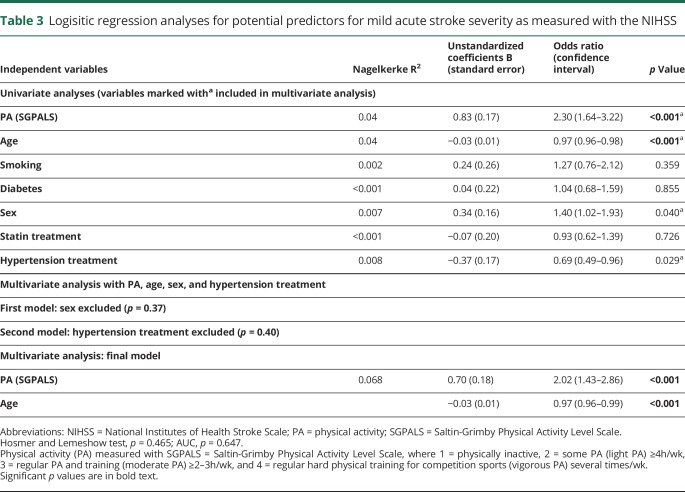
Logisitic regression analyses for potential predictors for mild acute stroke severity as measured with the NIHSS

## Discussion

This study suggests that prestroke PA combined with younger age is partially associated with milder stroke, which is in line with 2 previous studies of smaller populations (362 and 159 patients, respectively).^6,8^ The potential risk factors such as sex, smoking, diabetes, and protective treatments such as statin and hypertension treatment did not influence stroke severity in this study. In similar studies with some methodological differences including the definition of PA, stroke severity assessment, or assessment time, reduced stroke severity in patients who were physically active before stroke have been shown,^7,9,10^ although one study shows no effect.^11^ One subcategory of PA is exercise,^21^ often referred to in studies as PA with moderate to vigorous intensity, which excludes the patients performing light PA such as walking.^9,11^ However, 2 studies have shown that light PA also predicts less severe stroke.^8,10^ Hence, PA is an intervention known to prevent stroke, and the results of this study further indicate that prestroke PA also is associated with less severe stroke.

No dose dependency for intensity was found in this study, with no significant difference in stroke severity between the light PA group (walking or similar activity for at least 4 h/wk) and the moderate PA groups (training 2–3 h/wk). Two previous studies report dose dependency for duration of PA and mild stroke severity,^6,8^ while a third shows no dose dependency for frequency.^11^ Therefore, it is difficult to compare results between studies because frequency, intensity, and type of PA differ. However, these results presented here suggest that both light and moderate PAs are beneficial.

A majority of the patients were assessed as having a mild stroke, which could lead to motor, cognitive, or other functional deficits for the patient, even with a zero score on the NIHSS.^22^ Very few patients in this study had myocardial infarction or new stroke during their hospital stay; therefore, only descriptive statistics were produced for those outcomes (table 1). The length of hospital stay depends on many factors such as organization and comorbidity. Thus, regression analysis was not performed for the hospital stay outcome.

The strength of a registry-based study is the availability of previously collected variables from a large number of patients in clinical settings. Because the Swedish health care system is freely accessible to everyone, the Swedish quality registers gather information on all patients. The overall coverage ratio in the Riksstroke register is 90%. These factors form the basis for the generalizability of the results in this study.

The general limitations of a registry-based study such as this one is missing data, often found in specific variables (compared with clinical studies), and availability of predefined variables and measurements only, thereby reducing the number of available variables. A specific limitation of this study is the potentially limited utility of retrospective self-reported questions such as the SGPALS, although several studies have shown that self-report is a reproducible and reliable way to assess the activity level.^23–25^ The SGPALS has been validated as a simple indicator of inactive behavior and cardiovascular risk.^17,26^ Recall bias, where the individual's memory and the disease status can influence the recalling process, may give unreliable data.^27^ The self-reported assessments of PA are often used but have a larger variability compared with objective assessments. Although objective measurements are more accurate, the cost and feasibility can make the use difficult in larger populations.^28^

Some possible explanations for the benefits of prestroke PA for stroke severity could be reduction in inflammation or increase in the level of growth factors (which stimulates neurogenesis and angiogenesis).^29,30^ Whether these associations are truly caused by PA is not fully proven, nor the possible neuroprotective mechanisms, because most of such studies are conducted on animal models involving small sample sizes and constrained study design.^31^ Thus, there is a need for further research in this area to understand the possible mechanisms by which prestroke PA influences stroke severity.

The exploration of the link between prestroke PA and poststroke cognitive function has scope for future research. A recent study from Sweden showed an association between a high cardiovascular fitness in midlife women and decreased risk of dementia.^32^ Another possible research area is to investigate if and how prestroke PA has an effect on patients after stroke in a longer time perspective.

Physical inactivity should be monitored as a risk factor in clinical medical practice.^33^ Regular PA has been shown to reduce the risk of several noncommunicable diseases in addition to stroke, and increased PA is considered essential for maintaining good health. This can be achieved through education and recommendations regarding PA.^34^ However, the evidence of the neuroprotective effects of PA is conflicting, and studies with homogenous populations and standardized outcome analyses are needed.^35^ Health care should provide PA counseling and support for increased PA among individuals with risk factors.

This study shows that prestroke PA and younger age are partially associated with less severe acute stroke. Both light PA such as walking at least 4 h/wk and moderate PA 2–3 h/wk appear to be beneficial.

## Publication history

Received by *Neurology* January 4, 2018. Accepted in final form July 5, 2018.

## Glossary

ANOVA: analysis of variance
NIHSS: National Institutes of Health Stroke Scale
SGPALS: Saltin-Grimby Physical Activity Level Scale
PA: physical activity
